# 1149. Contribution of *S.pneumoniae* Serotypes in Adults with Invasive Pneumococcal Disease (IPD) and Pneumococcal Community-acquired Pneumonia (pCAP)

**DOI:** 10.1093/ofid/ofad500.990

**Published:** 2023-11-27

**Authors:** Kevin Apodaca, Lindsay Grant, Qi Yan, Christian Theilacker, Mohammad Ali, Michael W Pride, Bradford D Gessner, Luis Jodar

**Affiliations:** Pfizer Inc., New York, New York; Pfizer Inc., New York, New York; Pfizer Inc., New York, New York; Pfizer Inc., New York, New York; Pfizer Inc., New York, New York; Pfizer Vaccines, Pearl River, New York; Pfizer Biopharma Group, Collegeville, Pennsylvania; Pfizer Vaccines, Pearl River, New York

## Abstract

**Background:**

Invasive pneumococcal disease (IPD) serotype distribution has been used to infer the serotype distribution of pneumococcal community acquired pneumonia (pCAP) in countries where only IPD data exist. However, serotype distribution of IPD and pCAP may differ. We sought to identify differences in contribution of serotypes causing pCAP versus IPD.

**Methods:**

Eligible countries had serotype data available from adults for both IPD (determined by standard serotyping methods of cultured isolates) and pCAP (determined by serotype-specific urinary antigen detection (UAD) assays or culture for at least three years following PCV13 introduction into the pediatric national immunization program (NIP). Analyses were restricted to serotypes included in UAD assays. We further restricted analysis to serotypes not in PCV13 but detected by UAD assays (serotypes 2, 8, 9N, 10A, 11A, 12F, 15B/C, 17F, 20, 22F, and 33F), because PCV13 introduction into the NIPs has led to vaccine serotype disease reduction. The total positive of these serotypes was the denominator in the serotype-specific proportion calculations for both IPD and pCAP

**Results:**

We included seven countries. IPD serotype as well as pCAP distribution varied substantially across countries, while within countries, IPD and pCAP serotype distribution varied modestly (Figure1, Table 1) For the three most common pCAP serotypes across the seven countries, the absolute differences in the percent due to pCAP across countries were 19% (serotype 8), 10% (serotype 22F) and 10% (serotype 11A). For the same three serotypes, respectively, the absolute differences in the percent due to IPD across countries were 47%, 18%, and 17%. For these same three serotypes, the absolute differences in the percent due to pCAP vs, IPD within a country ranged from 1% to 14%, 1% to 6%, and 2% to 11%.

**Figure:**
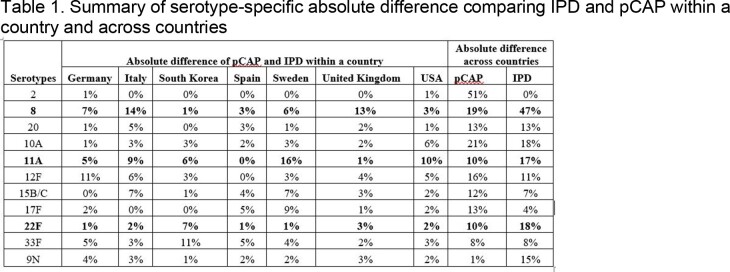

**Figure:**
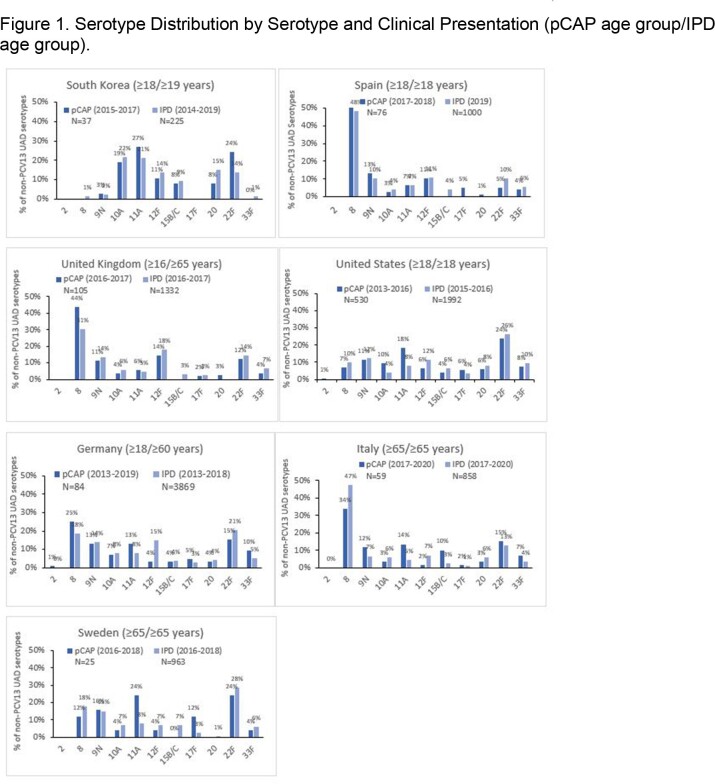

**Conclusion:**

pCAP and IPD serotype distribution were relatively aligned within countries. By contrast IPD and pCAP serotype distribution varied substantially across countries. Consequently, while the most accurate data for country-specific pneumococcal vaccine decision-making will come from combined IPD and CAP studies, countries without CAP data should rely on local IPD serotype distribution rather than CAP data from other countries.

**Disclosures:**

**Kevin Apodaca, MPH**, Pfizer Inc.: Stocks/Bonds **Lindsay Grant, PhD, MPH**, Pfizer Inc.: Stocks/Bonds **Qi Yan, PhD, MS**, Pfizer Inc: Stocks/Bonds **Christian Theilacker, MD, DTM&H**, Pfizer Inc: Stocks/Bonds **Mohammad Ali, PhD**, Pfizer Inc: Stocks/Bonds **Michael W. Pride, PhD**, Pfizer: Stocks/Bonds **Bradford D. Gessner, M.D., M.P.H.**, Pfizer: I am an employee of Pfizer|Pfizer: Stocks/Bonds **Luis Jodar, PhD**, Pfizer Inc: Stocks/Bonds

